# Leukostasis‐induced digital ischemia

**DOI:** 10.1002/jha2.666

**Published:** 2023-02-27

**Authors:** Alec Hasty, Mugdha Joshi, Dasom Lee, Gabriel N. Mannis

**Affiliations:** ^1^ Department of Medicine Stanford University Stanford California USA; ^2^ Division of Hematology Stanford University Stanford California USA

## 1 CASE PRESENTATION

A 70‐year‐old male presented with right‐sided facial droop and slurred speech. He was found to have a white blood count of 250 × 10^3^/μl with 81% peripheral blasts, and admitted to the medical intensive care unit for the management of leukostasis. In addition to a large left‐sided parietal infarct, other initial manifestations of leukostasis included non‐ST‐elevation myocardial infarction, splenic and renal infarcts, and possible pulmonary infarcts. He was initiated on leukapheresis and hydroxyurea, and after the result of peripheral blood flow cytometry showed acute myeloid leukemia (AML), he additionally received a single dose of cytarabine. On day 2 of admission, the patient noted increasing pain and cyanotic discoloration initially seen at the right toes and subsequently extended proximally up his right foot. Distal pulses were palpable, with normal ankle‐brachial index. Computed tomography angiography of the right lower extremity demonstrated a small distal dorsalis pedis artery thrombus. Vascular surgery was consulted and concluded the presentation was most consistent with microvascular ischemia that resulted from leukostasis. Severe thrombocytopenia, especially in the setting of multi‐system (including the central nervous system) involvement, ultimately precluded the use of anticoagulants. Without any surgical or pharmacological intervention, microvascular ischemia of his right foot and toes improved with treatment for AML. At the time of this writing, the distal digits had not yet undergone scheduled or auto‐amputation, and many of the other initial leukostasis manifestations were improving.

## 2 SUBJECT MATTER BACKGROUND

Leukostasis is a pathologic and clinical diagnosis resulting in end‐organ damage and is generally associated with a highly elevated number of circulating leukemic blasts [1, 2]. The mechanisms, though still under investigation, often are attributed to a combination of increased blood viscosity, decreased leukemic blast deformity, and leukemic blast‐endothelial cell interactions [1, 2, 3]. This more commonly involves the respiratory and central nervous systems and less frequently leads to cardiac, renal, or gastrointestinal complications [1, 3, 4]. Only seldom in the last two decades has leukostasis‐related acute limb ischemia been described [5, 6, 7, 8, 9]. This rare clinical manifestation is ultimately associated with high mortality and amputation risks [9]. It is a medical emergency that requires rapid stabilization of clinical presentation and cytoreduction of leukemic blasts.

## 3 CASE TAKEAWAY POINTS

**FIGURE 1:**
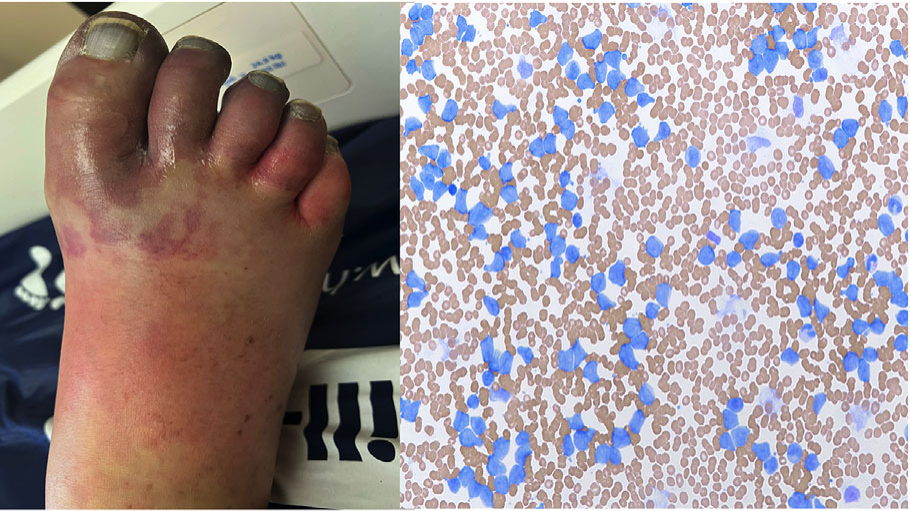
(A) Early acute limb ischemia changes of the right toes. (B) Peripheral smear with blasts during this leukostasis event.

In this case, the clinical presentation of leukostasis was widespread involving the brain, heart, lung, spleen, and kidney as well as microvascular ischemia of the right lower extremity. This case highlights the high morbidity of leukostasis, the need for prompt cytoreduction and treatment of underlying leukemia, as well as the importance of Figure 1 recognizing multi‐system and less common manifestations, such as acute limb ischemia.

## AUTHOR CONTRIBUTIONS

Alec Hasty, Mugdha Joshi, Dasom Lee, and Gabriel Mannis wrote and revised the manuscript.

## CONFLICT OF INTEREST STATEMENT

The authors declare no conflict of interest.

## FUNDING INFORMATION

The authors received no specific funding for this work.

## ETHICS STATEMENT

This original case report has not been previously published, submitted for publication elsewhere, and will not be published elsewhere without journal consent. Informed consent was obtained from the patient reported in this study.

## PATIENT CONSENT STATEMENT

Informed consent was obtained from the patient for this image report.

## Data Availability

Data sharing is not applicable to this article as there was no new data created or analyzed.
